# 831. Majocchi’s Granuloma – A Single Institution Retrospective Review

**DOI:** 10.1093/ofid/ofad500.876

**Published:** 2023-11-27

**Authors:** Carmen M Montagnon, Ryan B Khodadadi, Zachary A Yetmar, Emma F Johnson, Omar M Abu Saleh

**Affiliations:** Mayo Clinic Rochester, Rochester, Minnesota; Mayo Clinic, Rochester, Minnesota; Mayo Clinic, Rochester, Minnesota; Mayo Clinic Rochester, Rochester, Minnesota; Mayo Clinic Rochester, Rochester, Minnesota

## Abstract

**Background:**

Majocchi’s granuloma (MG) is an uncommon deep fungal folliculitis predominantly caused by dermatophytes. Available data regarding predisposing comorbidities/risk factors, clinical characteristics, offending microbiologic pathogens, diagnostics, pathologic findings, and treatment approaches has been inferred from historical cases. Given these limitations in the existing literature of MG, we sought to conduct a detailed review of our institutional experience with biopsy-confirmed cases of MG.

**Methods:**

Following IRB approval, we retrospectively identified and analyzed a multicenter cohort of adult patients diagnosed with MG between September 1992 and September 2022 at Mayo Clinic campuses (**Figure 1**). Data extracted included demographics, comorbid conditions, presenting clinical characteristics, diagnostic, microbiologic, and biopsy characteristics, treatment details, and outcomes.

**Figure 1**

**Figure:**
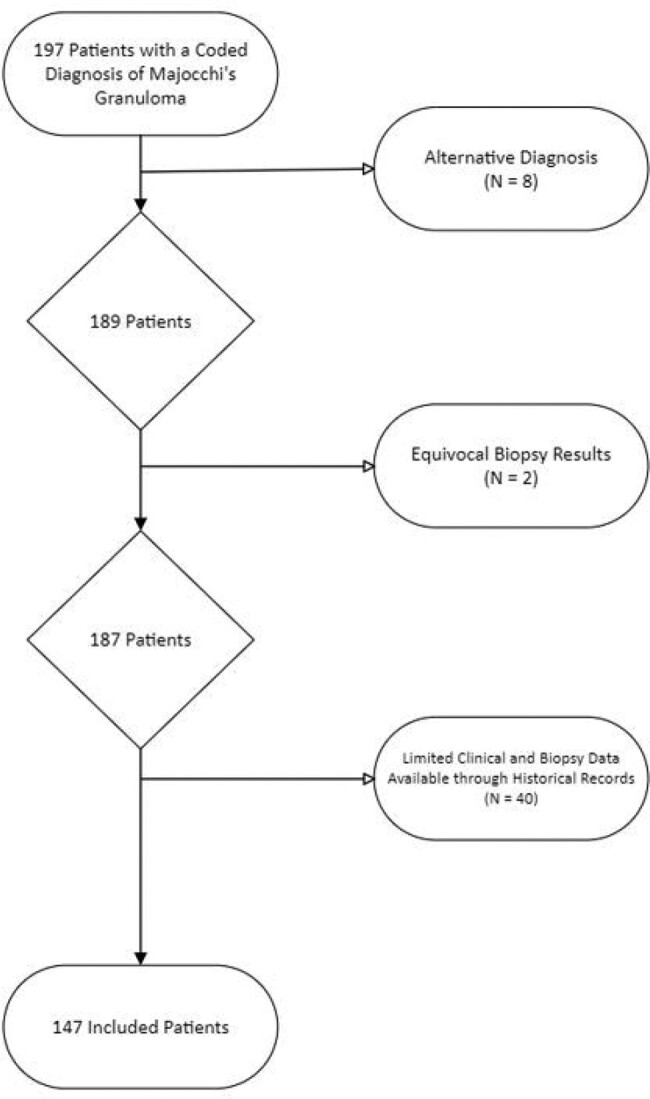

Flow Diagram show exclusion of patients from the final cohort.

**Results:**

A total of 147 patients with MG with the accompanying baseline characteristics met study criteria (**Table 1**). One-hundred and five patients were male with a median age of 55.6 years. Immunosuppressant and topical corticosteroid use were common prior to development of MG (**Table 1**). Dermatologic lesions and their sites of involvement did not differ based on the immune status of patients (**Table 2**). *Trichophyton rubrum* was the most common causative pathogen of MG, in addition to other dermatophytes and fungal organisms (**Table 3a**). Treatment duration for all prescribed agents was median 31.5 days. Oral terbinafine was the most frequently utilized agent and clinical resolution was achieved in 96.6% of cases (**Table 3b**).

**Figure:**
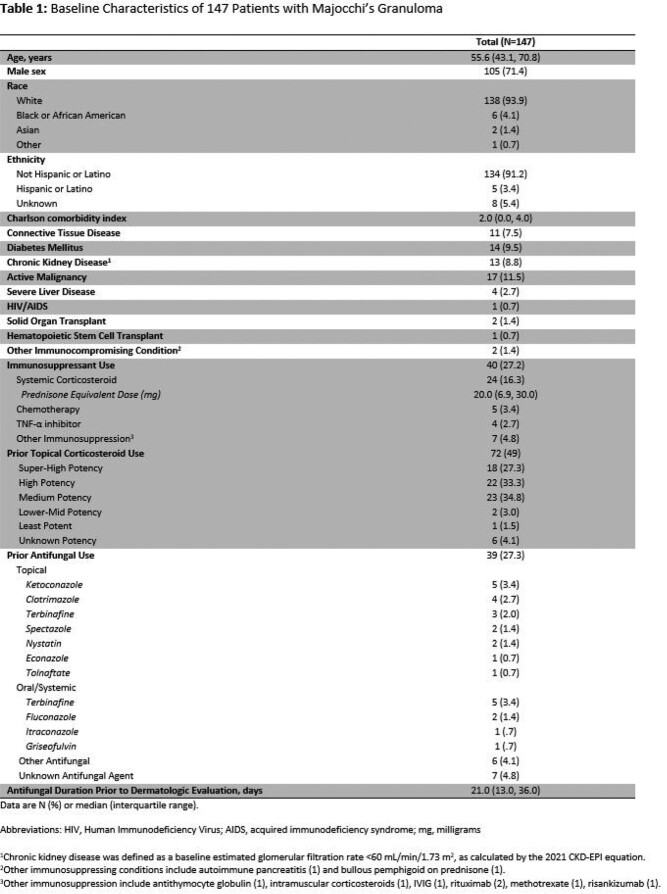

Baseline Characteristics of 147 Patients with Majocchi’s Granuloma

**Figure:**
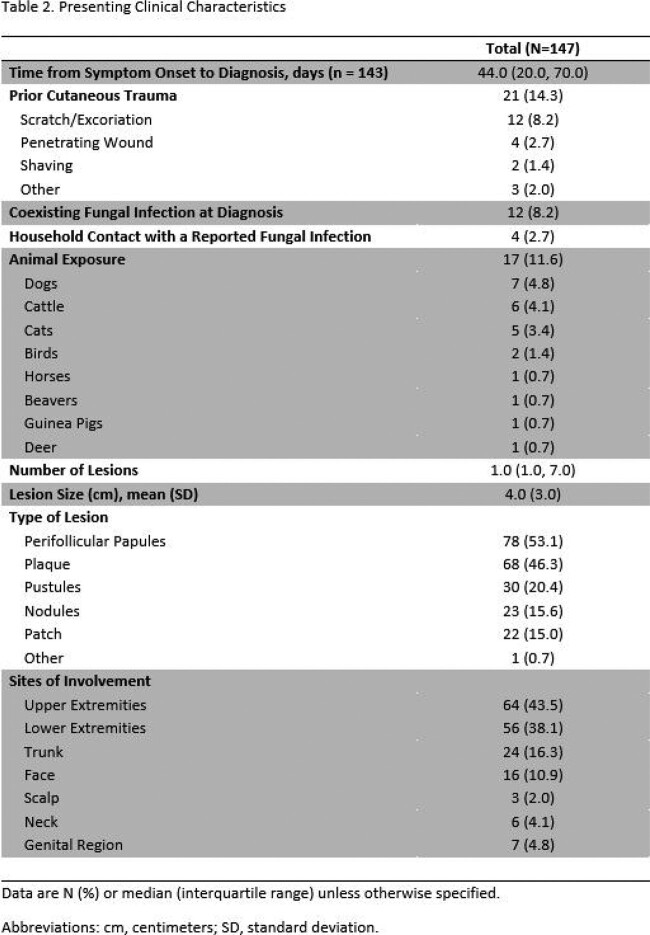

**Figure:**
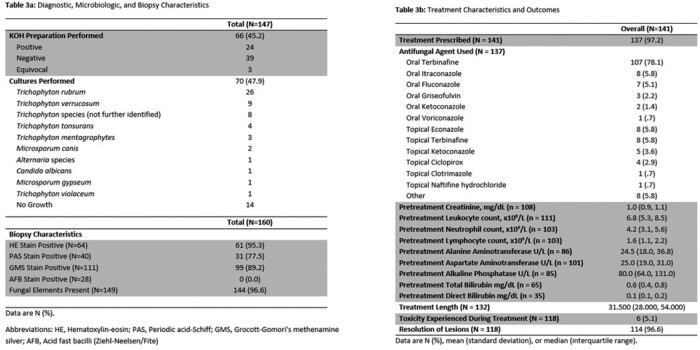

Diagnostic, Microbiologic, and Biopsy Characteristics, Treatment Characteristics and Outcomes

**Conclusion:**

While rare and clinically variable in presentation, the diagnosis of MG requires careful clinical examination and history-taking, histopathologic confirmation, and frequently fungal culture. Most cases of MG in our study responded well to a course of oral terbinafine at a median of 31.5 days of treatment.

**Disclosures:**

**All Authors**: No reported disclosures

